# Validation of potential candidate biomarkers of drug-induced nephrotoxicity and allodynia in medication-overuse headache

**DOI:** 10.1186/s10194-015-0559-8

**Published:** 2015-08-15

**Authors:** Elisa Bellei, Emanuela Monari, Stefania Bergamini, Aurora Cuoghi, Aldo Tomasi, Simona Guerzoni, Michela Ciccarese, Luigi Alberto Pini

**Affiliations:** Department of Diagnostic Medicine, Clinic and Public Health, Proteomic Lab, University of Modena and Reggio Emilia, Via del Pozzo 71, 41124 Modena, Italy; Headache and Drug Abuse Study Center, University of Modena and Reggio Emilia, Via del Pozzo 71, 41124 Modena, Italy

**Keywords:** Medication-Overuse Headache, Prostaglandin-H2 D-synthase, Cystatin-C, Alpha-1-microglobulin, Uromodulin, Urine, Western blot, Proteomics

## Abstract

**Background:**

Medication-overuse headache (MOH) is a chronic disorder that results from the overuse of analgesics drugs, triptans or other acute headache compounds. Although the exact mechanisms underlying MOH remain still unknown, several studies suggest that it may be associated with development of “central sensitization”, which may cause cutaneous allodynia (CA). Furthermore, the epidemiology of drug-induced disorders suggests that medication overuse could lead to nephrotoxicity. The aim of this work was to confirm and validate the results obtained from previous proteomics studies, in which we analyzed the urinary proteome of MOH patients in comparison with healthy non-abusers individuals.

**Methods:**

MOH patients were divided into groups on the basis of the drug abused: triptans, non-steroidal anti-inflammatory drugs (NSAIDs) and mixtures, (mainly containing indomethacin, paracetamol and, in some cases, caffeine). Healthy subjects, with a history of normal renal function, were used as controls. In this study, four proteins that were found differentially expressed in urine, and, on the basis of the literature review, resulted related to kidney diseases, were verified by Western Blot and Enzyme-linked Immunosorbent Assay (ELISA); Prostaglandin-H2 D-synthase (PTGDS), uromodulin (UROM), alpha-1-microglobulin (AMBP) and cystatin-C (CYSC).

**Results:**

Western blot analysis allowed to validate our previous proteomics data, confirming that all MOH patients groups show a significant over-excretion of urinary PTGDS, UROM, AMBP and CYSC (excluding triptans group for this latter), in comparison with controls. Moreover, the expression of PTGDS was further evaluated by ELISA. Also by this assay, a significant increase of PTGDS was observed in all MOH abusers, according to 2-DE and Western blot results.

**Conclusions:**

In this study, we confirmed previous findings concerning urinary proteins alterations in MOH patients, identified and demonstrated the over-expression of PTGDS, UROM, AMBP, and CYSC, particularly in NSAIDs and mixtures abusers. Over-expression of these proteins have been related to renal dysfunction and probably, PTGDS, to the development of CA. The detection and confirmation of this proteins pattern represent a promising tool for a better understanding of potential nephrotoxicity induced by drugs overuse and may enhance awareness related to the MOH-associated risks, even in absence of clinical symptoms.

## Background

A specific condition observed in chronic migraine patients, classified as medication-overuse headache (MOH) and characterized by the frequent intake of antimigraine drugs, is assumed to increase the frequency and intensity of headache [1]. MOH may complicate every type of headache and, in principle, all acute drugs used for headache treatment could cause MOH (i.e. ergotamine derivatives, triptans, simple and combined analgesics, barbiturates and opioids) [2]. Although the specific mechanisms leading to MOH remain still unknown, several studies suggest that MOH may involve amplification processes, including descending facilitation and “central sensitization”, and an increased excitability of spinal and medullary dorsal horn neurons resulting from a continuous input exerted by C-fiber nociceptors [3, 4]. This may lead to cutaneous allodynia (CA), a neurologic condition characterized by touch-evoked pain, elicited through ordinary non-nociceptive stimulation of the skin [5]. As a marker of central sensitization, allodynia has been proposed as a risk factor for progression to chronic migraine [6]. Recently, the development of MOH has been associated with long-lasting adaptive changes that occur within the peripheral and central nervous system. Preclinical studies have shown that repeated or continuous treatment with antimigraine drugs result in persistent up-regulation of neurotransmitters within the orofacial division of the trigeminal ganglia and in the development of CA in response to migraine triggers, even weeks after discontinuation of the antimigraine drug [7]. In our previous study we found elevated urinary levels of Prostaglandin-H2 D-synthase (PTGDS) in 3 MOH patient groups (triptans, NSAIDs and mixture abusers) in respect to healthy non-abusers individuals as control group [8]. Prostaglandin D2 is the most abundant prostanoid produced in the central nervous system of mammals, and is implicated in the modulation of neural functions, such as sleep induction, regulation of body temperature, nociception, pain responses and allodynia [9]. Some studies with animal models have demonstrated that prostaglandins play pivotal roles in central sensitization at spinal level, resulting in induction of hyperalgesia and CA (touch-evoked pain) [10]. Furthermore, elevated levels of PTGDS have been found in the serum of patients with renal impairment, so that the protein has even been suggested as a possible biochemical marker of renal insufficiency [11]. Therefore, PTGDS might contribute not only to the induction of allodynia [12], but also to the progression of chronic renal failure [13]. Based on the important functions assigned to PTGDS, the purpose of this study was the urinary quantification and validation in MOH abusers previously analyzed, by Western blotting and Enzyme-linked Immunosorbent Assay (ELISA). Moreover, in our previous works [8, 14] we identified, besides PTGDS, other proteins as potential biomarkers of nephrotoxicity, including Uromodulin (UROM), Alpha-1-microglobulin (AMBP) and Cystatin-C (CYTC). In recent years, proteomic researches have revealed numerous proteins as candidate biomarkers, but the lack of protein validation has represented a weakness for their application into clinical practice. The main purpose of the present work was to confirm and validate, by molecular biology techniques, proteins identified in earlier studies of our research group.

## Methods

### Subjects

Urine samples were taken from MOH patients, divided in 3 subgroups: triptans, NSAIDs and mixtures abusers. Moreover, urine of healthy non-abusers volunteers were collected and used as control. All patients groups and controls were matched for age and gender, and each subject gave informed consent to the study. Urinary routine parameters were measured in the clinical laboratory and resulted in the normal range. The exclusion criteria were renal insufficiency or kidney damage, ischemic heart disease, autoimmune disorders, oncologic or neurologic syndrome. The study received approval of the Ethical Committee of the University Hospital of Modena and was carried out in conformity with the Helsinki Declaration.

### Urine sample preparation

The second urine in the morning were collected into a sterile tube and centrifuged at 800 x g for 10 min at 4 °C, in order to remove cellular debris and contaminants. Then, urine samples were concentrated and desalted using filter devices with a 3 kDa MW cut-off (Millipore). The final total protein concentration was calculated by the Bradford method [15], using BSA as standard and rehydration buffer as blank.

### SDS-PAGE and two-dimensional gel electrophoresis

Sodium dodecyl sulphate-polyacrylamide gel electrophoresis (SDS-PAGE) was performed according to Laemmli’s procedure under reducing conditions, as previously reported [14]. In brief, 5 μg of total urine proteins for each group were mixed with the Laemmli sample buffer with the addition of β-mercaptoethanol as reducing agent. Samples were then boiled at 95 °C for 5 min and subsequently loaded onto 12 % SDS polyacrylamide gel. At the end of the electrophoresis run, gel were stained with Coomassie Blue G-250. Urine samples were also subjected to two-dimensional gel electrophoresis (2-DE) analysis, as previously described [8]. Briefly, 100 μg of total protein were subjected to first dimension separation (isoelectric focusing) using 17 cm IPG strip pH range 3-10 (Ready Strip™, Bio-Rad). Later, the second dimension separation was performed employing 8-16 % polyacrylamide gradient gel and the spot were visualized with silver nitrate staining protocol [16]. All gel images were acquired by a calibrated densitometer (GS800, Bio-Rad) and both the bands and the spot of interest were excised and stored at -20 °C until mass spectrometry (MS) analysis.

### Mass spectrometry protein identification

Protein bands and protein spot were “in-gel” digested as previously reported [17]. Briefly, they were first subjected to a step of de-staining (with acetonitrile for protein bands and with a solution of potassium hexacyano-ferrate(III)/sodium thiosulphate for protein spot, respectively). In the next step, both samples were reduced with dithiotreitol and alkylated with iodoacetamide, followed by trypsin digestion at 37 °C overnight. The obtained peptides were extracted by a two-phase procedure, first with acetonitrile/ammonium bicarbonate and then using formic acid. Finally, the pooled peptides were concentrated in a vacuum dryer before MS analysis by a Nano LC-CHIP-MS system, composed of the 6520 ESI-Q-ToF coupled with a Nano HPLC-Chip microfluidic device (Agilent Technologies Inc., CA, USA), as previously described in detail [17]. The MASCOT search engine (version 2.4) was used for peptide sequence searching against the UniProt database, setting the following restrictions: *Homo sapiens* taxonomy (Human), parent ion tolerance ±20 ppm, MS/MS error tolerance ±0.1 Da, alkylation of cysteine residues (fixed modifications), oxidation of methionine (variable modifications), and two potentially missed trypsin cleavages. The highest score hits among MASCOT search results were selected. Protein identification was repeated at least once, using band/spot cut from replicated gel.

### Western blotting analysis

A total of 1.5 μg urine proteins were separated on 12 % SDS-PAGE and blotted onto nitrocellulose membranes, that were first blocked with 5 % non-fat milk and subsequently incubated overnight at 4 °C with the following primary antibodies (all from Abcam, Cambridge, UK): anti-Prostaglandin D Synthase (rabbit polyclonal, 1:500); anti-Uromucoid (rabbit polyclonal, 1:500); anti-Alpha-1-microglobulin (rabbit monoclonal, 1:1000); anti-Cystatin C (rabbit monoclonal, 1:500). Membranes were then incubated with a solution containing 1:2000 dilution of horseradish peroxidase (HRP)-conjugated goat anti-rabbit secondary antibody (DakoCytomation, Denmark). Target bands were visualized using a mix of peroxidase solution plus a luminol enhancer solution (WesternSure™ PREMIUM Chemiluminescent substrate). Results acquisition and band densitometric analysis (represented by arbitrary units, AU), were performed using the C-DiGit® Blot Scanner (LI-COR Biosciences, NE, USA) and the QuantityOne image analysis software (Bio-Rad). Human serum sample was used as positive (or negative) control.

### Measurement of PTGDS by ELISA

Immunoreactive PTGDS was determined by ELISA using a commercially available kit (BioVendor, NC, USA), on the basis of the manufacturer’s instructions. Briefly, urine samples were diluted 100-fold with dilution buffer and then incubated for 1 h at room temperature with polyclonal anti-human L-PTGDS antibody immobilized to the surface of the plate wells. After three wash, 100 mL of conjugate solution (anti-PTGDS conjugated with horseradish peroxidase, HRP) were added and the plate incubated for 1 h at room temperature. Following 3 washing steps, the remaining HRP conjugate was allowed to react with the substrate solution (tetramethylbenzidine). Finally, the reaction was stopped by the addition of acidic solution and absorbance of the resulting yellow product was measured at λ 450 and 620 nm, using a microplate reader (Multiscan FC, Thermo Scientific, MA, USA). PTGDS concentrations were determined from a standard curve generated by the standards supplied with the kit.

### Data analysis

A statistical analysis of ELISA results (for urinary PTGDS), and of Western blot signal values (obtained from all proteins tested in each different group), was done with the Student *t*-test. A p-value <0.05 was considered as statistically significant. All data reported in Figs. 3 and 4 are provided as mean ± standard deviation (SD).

## Results

### SDS-PAGE, 2-DE and image analysis

**Fig. 1:**
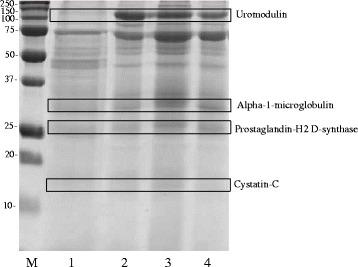
SDS-PAGE. The protein profiles were obtained from urine of healthy controls (lane 1), triptans (lane 2), NSAIDs (lane 3), and mixtures abusers (lane 4). M = molecular weight marker ladder (DualColor, Bio-Rad). In box are evidenced the bands related to the investigated proteins for each group. Poliacrylamide gel 12 % and Coomassie blue staining

**Fig. 2:**
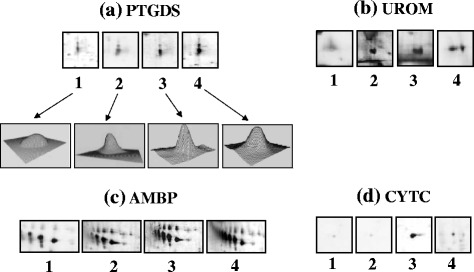
Magnified spot section from 2D gel. Comparison of protein spot obtained by 2-DE analysis of urine samples from healthy controls (1), triptans (2), NSAIDs (3), and mixtures abusers (4), for the four examined proteins: Prostaglandin-H2 D-synthase (**a**), Uromodulin (**b**), Aplha-1-microglobulin (**c**) and Cystatin-C (**d**). For PTGDS protein was also reported the peack illustrating its relative abundance, obtained for each group by the PDQuest software. First dimension was made with IPG strips 17 cm NL, pH 3-10; the second dimension was performed using 8-16 % polyacrylamide gradient gels; 0.2 % silver nitrate was used for gel staining

Urine proteins were first separated according to their molecular weight by SDS-PAGE (Fig. 1) and gel images were acquired by a calibrated densitometer (GS800, Bio-Rad). The bands enclosed in rectangles were cut from each lane (corresponding to every group of patients and controls) and were subjected to MS analysis. As evident in Fig. 1, the result was the identification of the following proteins: UROM, expressed as a very intense band in all MOH patients (lane 2 = triptans, lane 3 = NSAIDs and lane 4 = mixtures abusers) respect to controls (lane 1); AMBP, particularly evident in NSAIDs group; PTGDS, an intensive band visible in NSAIDs and mixtures groups, which was much less observable in triptans abusers and even more in controls, and CYTC, with a perceptible band in NSAIDs group. To strengthen these results, we analyzed the same samples by 2-DE. Isolated and magnified differentially expressed spots obtained from 2D gels are reported in Fig. 2. The results overlapped those obtained by SDS-PAGE. In fact, analyzing the spot staining intensity by the PDQuest image analysis software (version 7.3.1, Bio-Rad), PTGDS was significantly over-excreted in NSAIDs (A3), mixtures (A4) and triptans groups (A2) in comparison to controls (A1); UROM resulted over-expressed in triptans (B2), NSAIDs (B3) and mixtures abusers (B4), compared to controls (B1); AMBP spots were significantly increased only in NSAIDs (C3) and mixtures abusers (C4) respect to triptans (C2) and controls (C1); finally, CYTC resulted particularly elevated in NSAIDs abusers (D3). Furthermore, we illustrated the 3D views of PTGDS protein spot, developed with the PDQuest software, in order to provide a clearer vision of its expression change in the examined groups.

### MS protein identification

MS analysis was performed using an Electrospray-Quadrupole-Time of Flight (ESI-Q-ToF) mass spectrometer (Agilent Technologies, CA, USA). Protein identification was achieved using the Agilent MassHunter Workstation software (version B.02.00) and the search was conducted by MASCOT search engine (version 2.4) against the UniProt database. During MASCOT search, the significant threshold was set up to maintain the False discovery Rate (FDR) below 1 %. The identification was done in duplicate, cutting bands and spot from replicate gels. The results obtained for each identified protein are listed in Table 1. Column 1 lists the protein entry names according to the UniProt knowledge database, while the others columns show the MS analysis data, such as the ion scores (column 2), expressed as the probability that the observed match between the experimental data and the database sequence could be due to a random event; queries (column 3), that is the total number of peptides that matched the identified proteins and the significant matches; the total number of sequences and the number of significant sequences (column 4) and the sequence coverage, namely the percentage of amino acids sequenced (final column).

**Table 1:** MS protein identification by ESI-Q-ToF-MS

Protein name^a^	Score^b^	N° matches/sign.matches^c^	N° seq./sign.seq.^d^	Seq. cov.^e^
PTGDS	158	25/14	3/2	33 %
UROM	86	18/13	3/2	47 %
AMBP	201	21/12	4/3	32 %
CYTC	71	16/10	3/3	52 %

^a^Protein entry name (UniProt knowledge database)

^b^The highest scores obtained using MASCOT search engine

^c^The total number of peptides matched and the significant matches

^d^The total number of sequences and the number of significant sequences

^e^Sequence coverage: the percentage of amino acids sequenced for the detected protein

### Western blotting

**Fig. 3:**
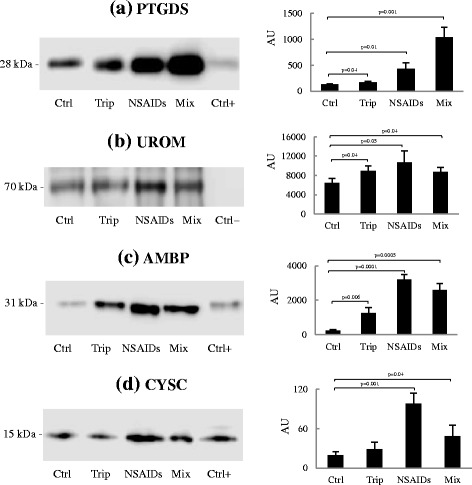
Protein expression by Western blot. The analysis for each protein was conducted on urine samples from the four groups. Serum sample was used as positive (or negative) control. The histograms show the quantitative representation of PTGDS (**a**), UROM (**b**), AMBP (**c**) and CYSC (**d**) obtained by densitometric analysis with QuantityOne image analysis software (the data represent mean ± SD). Ctrl, healthy control group; Trip, triptans abusers; NSAIDs abusers; Mix, mixtures abusers

In order to validate the results of electrophoresis (SDS-PAGE e 2-DE) and precisely verify the identity of proteins inferred from ESI-Q-ToF-MS analysis, we evaluated the levels of PTGDS, UROM, AMBP and CYTC by Western blot. The analysis was conducted with ten urine samples for each group. As shown in Fig. 3a, PTGDS protein was detected in all urine samples from every group, and precisely was found to have higher levels in mixtures (*p* = 0.001) and NSAIDs groups (*p* = 0.01), respect to triptans abusers (*p* = 0.04), when compared *vs* healthy controls, similarly to the results of 2-DE analysis. UROM (Fig. 3b) showed a marked signal at 70 kDa and a significant increase in all MOH patients compared to control group. During Western Blot analysis, we used human serum sample as control; regarding UROM, since it is exclusively produced in the kidney and secreted into the urine via proteolytic cleavage, no band was observed at UROM molecular weight. Therefore, serum sample can be considered as negative control, while it represents a positive control for PTGDS, AMBP and CYSC, that showed a clear signal; these 3 proteins, present in serum, are filtered by the kidney and excreted in urine. AMBP signal (Fig. 3c) was highly significant in all MOH abusers, particularly in NSAIDs and mixtures groups (*p* = 0.0001 and *p* = 0.0005, respectively) and also in triptans (*p* = 0.006) compared to controls. Finally, the increase of CYTC (Fig. 3d) showed its maximum signal in NSAIDs and mixtures abusers (*p* = 0.001 and *p* = 0.04 *vs* control group), while triptans group showed no significativity.

### ELISA results

**Fig. 4:**
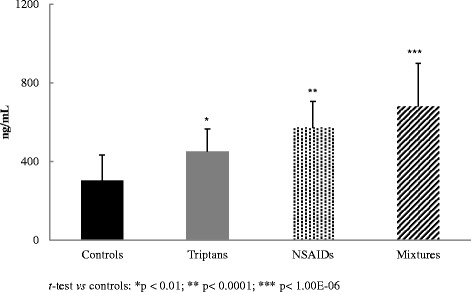
Immunoreactivity of PTGDS by ELISA assay. Results are expressed as mean ± SD. Significant differences were assessed by unpaired Student’t *t*-test (*p < 0.01; **p < 0.0001; ***p < 1.00E-06 vs control group)

The expression level of PTGDS was estimated by ELISA assay (Fig. 4). When compared to control subjects, a significant increase in PTGDS immunoreactivity was observed in all MOH patients groups. Particularly, PTGDS level was highly significant in mixtures (681 ± 218 ng/mL, p < 1.00E-06) and NSAIDs abusers (572 ± 135 ng/mL, p < 0.0001) in respect to triptans abusers (450 ± 116 ng/mL, p < 0.01), when compared to control group (303 ± 130 ng/mL). These results are fully consistent with the data from 2-DE (Fig. 2-a) and Western blot analysis (Fig. 3a). The measured values of urinary PTGDS fell on the linear portion of the ELISA kit standard curve.

## Discussion

In the present study we carried out additional analysis, such as Western blot and ELISA assays, to validate our previous findings aimed to discover early biomarkers of drug-induced nephrotoxicity in MOH [8, 14], and to enhance its accuracy of prediction. Among the differentially expressed proteins previously identified, our study focused on UROM, AMBP and CYSC, since an ample literature provides evidence of their involvement in renal damage and nephropathy. The special role played by PTGDS, which is implicated in pain onset (particularly CA), was also investigated [13]. CA is defined as pain in response to non-nociceptive thermal and mechanical stimuli applied to normal skin, a very uncomfortable heightened sensitivity to touch [18, 19]. Some studies indicated that up to 80 % of migraine patients reported CA during an acute attack [6] or abnormal sensitivity of extracranial areas [20]; others showed that most migraine patients exhibit CA inside and outside their pain-referred areas when examined during a fully developed migraine attack [21]. CA in migraine is a clinical manifestation of central nervous system sensitization, and consequently several chronic pain syndromes and mood disorders are comorbid with migraine [22]. Given the complexity of pain and its arduous and not particularly effective treatment, there is an important need to define who is susceptible to pain hypersensitivity, as well as to discover new molecules and mechanisms finalized to the identification of new therapeutic interventions with greater efficacy. Studies has been recently carried out to prove epigenetics role in the causation of chronic pain [23], trying to clarify a pain-specific protein interaction network [24, 25]. In the present study we focused on PTGDS, also known as β-trace protein, a lipocalin-type prostaglandin that is responsible for the conversion of prostaglandin H_2_ (PGH_2_) into prostaglandin D_2_ (PGD_2_), in the presence of sulfhydryl compounds [26]. PTGDS is actively produced in a variety of tissues and is involved in numerous physiological and pathological functions, such as vasodilatation, inhibition of platelet aggregation and nitric oxide release; moreover, it is a potent endogenous nociceptive modulator [9]. Western blot analysis (Fig. 3a) and ELISA test (Fig. 4) lead to the verification and validation of the proteomic data (Figs. 1 and 2-a), confirming that the MOH patients examined in this work show an over-expression of urinary PTGDS, especially NSAIDs and mixtures abusers, when compared with triptans group and more *vs* healthy controls. On the basis of these results, a clear indication arises directing to the involvement of PTGDS in the manifestation of CA, by decreasing pain threshold, as verified in MOH abusers and in migraineurs patients recruited in a previous study [27]. With the abuse of antimigraine drugs, migraineurs patients may develop MOH, a frequent and disabling condition characterized by increased headache frequency and intensity, inefficacy of medications and development of drug dependence [7]. Pain progression has been evaluated in MOH patients, suggesting the presence of a global alteration in the processing of noxious stimuli throughout the pain matrix and the occurrence of significant functional changes in the lateral pain pathway [28]. Only by understanding the molecular circuits complexity and the substances mediating pain, the development of increasingly specific tools for the identification of new markers, will be possible. In our study, MOH patients, showing high levels of PTGDS, suffer pain growth and progression, suggesting that PTGDS is indeed a potential urinary biomarker indicating CA development. Different types of prostaglandins play a key role in important physiological conditions, such as renal function and development. PTGDS is involved in the advancement of kidney diseases, and has been proposed in the past years as a potential diagnostic marker for renal injury [29]. Recently, animal studies have shown that the urinary excretion of PTGDS may predict the development of proteinuria and renal injury [30]. In our study, an elevated PTGDS level was determined in the urine of MOH patients (particularly in NSAIDs and mixture abusers) (Figs. 2-a, 3a and 4), suggesting the importance of monitoring MOH patients renal function that, at its turn, will enable the prevention of the drug-induced nephrotoxicity.

Other proteins involved in renal dysfunction, also tested in this study, were UROM, AMBP and CYSC. UROM (Tamm-Horsfall glycoprotein) is the most abundant protein excreted in the urine under physiological conditions, being exclusively synthesized by the cells of the thick ascending limb and early distal convoluted tubule of the kidney. UROM is produced in the endoplasmic reticulum, shuttled to the apical cell membrane, and released into the urine by proteolytic cleavage [31]. UROM has been known for more than 50 years and since its discovery several researches have been conducted, revealing novel roles for this protein [32]. Recently, genome-wide association studies identified UROM as a risk factor for chronic kidney disease (CKD) and hypertension, suggesting that the urinary level of UROM represents a useful biomarker for the development and progression of CDK [33, 34]. In our study, by Western blot analysis we confirmed a significant over-excretion of UROM (Fig. 3b) in all MOH patients compared to the control group; the same was also observed for AMBP (Fig. 3c). AMBP is a low molecular weight protein, also called protein HC, which is readily filtered by the glomerulus and reabsorbed and catabolised by the proximal tubular cells. Therefore, the presence of AMBP in urine is indicative of reduced resorptive capacity of the proximal tubule [35]; consequently, the urinary concentration of AMBP, which is stable at low pH, designate this protein as a useful marker of proximal tubular abnormalities and chronic asymptomatic renal tubular dysfunction [36]. Moreover, urinary AMBP can be considered as a useful marker for the early detection and monitoring of nephropathy in type 2 diabetes [37]. Finally, we found a significantly increased level of CYSC in NSAIDs and mixtures abusers, but not in triptans abusers (Fig. 3d). Also CYSC has been used for many years as a clinical marker of kidney function [38]. This 15-kDa cysteine proteinase inhibitor is produced by all nucleated cells at a constant rate and constitutively secreted shortly after its synthesis. Following glomerular filtration, CYSC is reabsorbed by the proximal tubular cells, where it is almost completely catabolized, while the remaining uncatabolized protein is eliminated in the urine [39]. Thus, normal urinary CYSC concentration is very low, whereas in case of tubular diseases CYSC degradation is reduced, leading to an increase in its urinary elimination. Furthermore, a recent study reported that urinary CYSC levels and tubular proteinuria may predict the progression of type 2 diabetic nephropathy [40]. Accumulating evidence suggests CYSC as a reliable biomarker and predictor of impaired renal function, in particular of tubular damage [41]. In summary, we have now firmly established that PTGDS, UROM, AMBP and CYSC are proteins over-excreted in the urine of MOH patients, especially in NSAIDs and mixtures abusers, compared to healthy non-abusers individuals.

The debate on the association between nonphenacetin-containing combined analgesics and renal disease has been going on for a long time. Some years ago, an international ad hoc peer-reviewed committee of scientists concluded that there is no sufficient evidence to associate nonphenacetin combined analgesics with nephropathy [42]. A population-based case–control study with incident cases of end-stage renal disease (ESRD) demonstrated that the use of a high cumulative lifetime dose (3^rd^ tertile) of analgesics up to five years before dialysis was not associated with ESRD [43]. Others case-controls studies have shown that caffeine-containing analgesics are associated with analgesic nephropathy (odds ratio = 4.9, 95 % CI 2.3 to 10.3) [44]. In the series observed in our studies, we did not register any case of clinical impairment of renal functions. The main NSAIDs used were indomethacin, paracetamol and, in some cases, compounds containing caffeine. However, if caffeine produces nephrotoxicity on its own, or increases analgesics-related nephrotoxicity is yet to be established [44]. In literature there is a lack of definite data regarding causative analgesics, including those concerning paracetamol. Hence, patients should not be withheld for paracetamol, an effective and commonly recommended agent, for fear of worsening renal function [45], but, at the same time, an increasing universal awareness about rational use of analgesics is important for MOH prevention.

## Conclusions

MOH has a prevalence of 1-2 % in the general population worldwide and it is likely to be the most costly neurological disorder known [46]. Even more significantly, MOH has similarities with traditional drug addiction. Nonetheless, there is a lack of research into awareness, education and prevention of MOH [47, 48]. With the present work we firmly confirmed and strengthen our previous findings regarding the possibility of drug-induced nephrotoxicity in MOH patients, particularly in the case of NSAIDs and mixtures abuse. These results contribute to emphasize the importance in providing educational and preventive strategies concerning the risks linked to MOH, such as the probability of developing renal injuries. Therefore, the proteins under our scrutiny may represent a reliable and distinctive panel of prospective early target of kidney dysfunctions, useful to monitor over time renal function of MOH abusers, recognizing patients prone to progress toward nephropathy. Accordingly, these findings could enhance the awareness about the risks associated to MOH, helping to reduce morbidity. Moreover, the present results on PTGDS may be useful to provide a common target for advanced study, aimed to analyze pain mechanisms and pathways at the molecular level, particularly in the case of CA. The increase of urinary PTGDS observed also in patients taking triptans could be an early indicator of a nervous system driven up-regulation associated to chronic pain, as in the case of MOH. Even if there are no conclusive data showing a direct impact of NSAIDs on kidney functions in headache patients, these findings could represent an initial marker linked to a specific type of pain, such as CA.

## Abbreviations

AMBP: Alpha-1-microglobulin
CA: Cutaneous allodynia
CYTC: Cystatin-C
2-DE: Two-dimensional gel electrophoresis
MS: Mass spectrometry
PTGDS: Prostaglandin-H2 D-synthase
UROM: Uromodulin
